# Evaluation of plasma IL-21 as a potential biomarker for type 1 diabetes progression

**DOI:** 10.3389/fimmu.2023.1157265

**Published:** 2023-06-21

**Authors:** Anna-Mari Schroderus, Josh Poorbaugh, Samantha McElyea, Stephanie Beasley, Lin Zhang, Kirsti Näntö-Salonen, Reeta Rintamäki, Jussi Pihlajamäki, Mikael Knip, Riitta Veijola, Jorma Toppari, Jorma Ilonen, Robert J. Benschop, Tuure Kinnunen

**Affiliations:** ^1^ Department of Clinical Microbiology, Institute of Clinical Medicine, University of Eastern Finland, Kuopio, Finland; ^2^ Eli Lilly and Company, Indianapolis, IN, United States; ^3^ Department of Pediatrics, Turku University Hospital, Turku, Finland; ^4^ Department of Medicine, Endocrinology and Clinical Nutrition, Kuopio University Hospital, Kuopio, Finland; ^5^ Institute of Public Health and Clinical Nutrition, University of Eastern Finland, Kuopio, Finland; ^6^ Tampere Center for Child Health Research, Tampere University Hospital, Tampere, Finland; ^7^ Pediatric Research Center, New Children’s Hospital, Helsinki University Hospital, Helsinki, Finland; ^8^ Research Program for Clinical and Molecular Metabolism, Faculty of Medicine, University of Helsinki, Helsinki, Finland; ^9^ PEDEGO Research Unit, Department of Pediatrics, Medical Research Center, Oulu University Hospital and University of Oulu, Oulu, Finland; ^10^ Institute of Biomedicine, Research Centre for Integrative Physiology and Pharmacology, Centre for Population Health Research, University of Turku, Turku, Finland; ^11^ Immunogenetics Laboratory, Institute of Biomedicine, University of Turku, Turku, Finland; ^12^ ISLAB Laboratory Centre, Kuopio, Finland

**Keywords:** autoimmunity, autoimmune diseases, interleukin-21, IL-21, human, type 1 diabetes

## Abstract

IL-21 is a multifunctional cytokine linked with the pathophysiology of several autoimmune diseases, including type 1 diabetes. In this study, our aim was to examine plasma IL-21 levels in individuals at different stages of type 1 diabetes progression. We measured plasma IL-21 levels, as well as levels of other key pro-inflammatory cytokines (IL-17A, TNF-α and IL-6), from 37 adults with established type 1 diabetes and 46 healthy age-matched adult controls, as well as from 53 children with newly diagnosed type 1 diabetes, 48 at-risk children positive for type 1 diabetes-associated autoantibodies and 123 healthy age-matched pediatric controls using the ultrasensitive Quanterix SiMoA technology. Adults with established type 1 diabetes had higher plasma IL-21 levels compared to healthy controls. However, the plasma IL-21 levels showed no statistically significant correlation with clinical variables, such as BMI, C-peptide, HbA1c, or hsCRP levels, evaluated in parallel. In children, plasma IL-21 levels were almost ten times higher than in adults. However, no significant differences in plasma IL-21 levels were detected between healthy children, autoantibody-positive at-risk children, and children with newly diagnosed type 1 diabetes. In conclusion, plasma IL-21 levels in adults with established type 1 diabetes were increased, which may be associated with autoimmunity. The physiologically high plasma IL-21 levels in children may, however, reduce the potential of IL-21 as a biomarker for autoimmunity in pediatric subjects.

## 1 Introduction

Type 1 diabetes (T1D) is a chronic autoimmune disease, in which autoreactive T cells mediate the progressive destruction of insulin-producing beta cells in the pancreas. T1D is preceded by a preclinical phase of variable length, during which circulating autoantibodies (AAb) to islet autoantigens are almost invariably detected (1). It is well-established that T cells play a central role in the T1D disease process (2). However, the exact T-cell phenotypes or immune pathways involved remain largely elusive. Recently, the interleukin-21 (IL-21) pathway has gained attention, as it has been associated with the development of T1D both in murine models (3) as well as in human studies (4–6).

IL-21 is a pleiotropic cytokine with multiple functions. It is a hallmark cytokine of CD4^+^CXCR5^+^ T follicular helper (Tfh) cells (7, 8). In addition, it is produced by Th17 cells (7) and a recently identified population, coined T peripheral helper (Tph) cells (9, 10). IL-21 has a fundamental role in B-cell helper functions. In order to activate, expand and produce antibodies, B cells are largely dependent on the help provided by IL-21-producing Tfh cells in lymph nodes (7, 8), and possibly also by Tph cells within inflamed tissues (10). Importantly, IL-21 is also known to support the effector functions and cytotoxicity of CD8^+^ T cells (7). IL-21 produced by CD4^+^ T cells may therefore contribute to the T1D disease process also by activating autoreactive CD8^+^ T cells in the pancreas (11–13).

Interestingly, an expansion of circulating Tfh and Tph cells and increased IL-21 production by T cells has been observed both in children with newly diagnosed T1D (6, 14), as well as in adults with established T1D (4, 5). Moreover, Th17 cells, another potential source of IL-21, appear to be increased both in the blood and pancreatic lymph nodes of patients with T1D (15, 16). These findings implicate the IL-21 pathway to be an appealing candidate for immunotherapy of T1D. Promisingly, in a recently completed phase 2 trial, an anti-IL-21 antibody combined with liraglutide was shown to preserve beta-cell function in patients with recent-onset T1D (17).

Given the importance of IL-21 for autoimmunity, blood IL-21 levels could serve as a potential biomarker of disease activity in autoimmune disorders. Indeed, in some autoimmune diseases, such as Sjögren’s syndrome (18) and systemic lupus erythematosus (SLE) (19), plasma and/or serum IL-21 levels are elevated in patients. Recently, limitations in the specificity and sensitivity of commercially available human IL-21 ELISA kits have been identified, and as a result, a new ultrasensitive assay for detecting IL-21 based on the Quanterix SiMoA (Single Molecule Array) technology was developed (20). With this assay, it was demonstrated that IL-21 concentrations in plasma and serum are considerably lower than previously published data using less sensitive and less specific assays suggested. Importantly, higher plasma IL-21 levels in patients with Sjögren’s syndrome and SLE compared to healthy individuals were also validated with this ultrasensitive assay (20).

To our knowledge, plasma IL-21 levels have previously been investigated in a limited number of studies related to T1D, all of which have suggested that patients with T1D (21, 22) and AAb^+^ at-risk children (23) have higher IL-21 levels in blood than controls. In this study, we analyzed plasma IL-21, as well as levels of other key pro-inflammatory cytokines (IL-17A, TNF-α and IL-6) with the ultrasensitive Quanterix SiMoA technology at different stages of T1D development, utilizing samples from cross-sectional cohorts of adults with established T1D, children with newly diagnosed T1D, and AAb^+^ at-risk children together with age-matched controls.

## 2 Materials and methods

### 2.1 Study subjects

The adult cohort comprised 37 patients with established T1D and 46 healthy age-matched controls (**Table 1**). The pediatric cohort consisted of 53 children with newly diagnosed T1D (0 to 7 days after clinical diagnosis), 48 autoantibody-positive (AAb^+^) at-risk children, and 123 healthy control children who were autoantibody-negative and age-matched with the T1D and AAb^+^ cases (**Table 2**). The AAb^+^ and healthy control children participated in the Finnish Type 1 Diabetes Prediction and Prevention (DIPP) follow-up study and carried HLA genotypes associated with increased risk for T1D. Autoantibody-positivity was analyzed, as previously described (24). Autoantibody-positivity was defined based on the presence of one or more biochemical autoantibodies (insulin autoantibodies [IAA], insulinoma-associated-2 antibodies [IA-2A] and glutamic acid decarboxylase antibodies [GADA]).

**Table 1:** Characteristics of the adult cohort.

Study group	Healthy controls	T1D patients
**n**	46	37
**Age (years), mean ± SD**	26.1 ± 4.3	26.6 ± 6.4
**Age range (years)**	20–38	18–39
**Disease duration (years), mean ± SD**	N/A^*^	11.7 ± 8.1
**Disease duration range (years)**	N/A	0–36
**Male**	40% (20/46)	57% (21/37)
**Female**	60% (26/46)	43% (16/37)
Clinical variables:		
**HbA1c (mmol/mol), mean ± SD**	ND^†^	64.6 ± 13.7 (n=32)
**C-peptide (nmol/L), mean ± SD**	ND	0.11± 0.17
**BMI (kg/m^2^), mean ± SD**	ND	26.6 ± 5.1 (n=29)
**hsCRP (mg/L), mean ± SD**	1.31± 1.96	3.15 ± 5.14

*not applicable, ^†^not determined.

**Table 2:** Characteristics of the pediatric cohort.

Study group	Healthy children	AAb^+^ at-risk children	Newly diagnosed T1D patients
**n**	123	48	53
**Age (years), mean ± SD**	8.9 ± 4.0	8.6 ± 4.7	8.5 ± 3.8
**Age range (years)**	2.0–15.7	2.0–17.2	2.4–17.5
**Male**	63% (77/123)	54% (26/48)	51% (27/53)
**Female**	37% (46/123)	46% (22/48)	49% (26/53)
Progressors^*^	N/A^†^	52% (25/48)	N/A
**Time (years) from sampling to clinical T1D, mean ± SD**	N/A	2.6 ± 1.4	N/A
**Time (years) from birth to clinical T1D, mean ± SD**	N/A	10.0 ± 4.1	N/A
Autoantibodies:			
**GADA**	N/A	63% (30/48)	68% (36/53)
**IA-2A**	N/A	67% (32/48)	75% (40/53)
**IAA**	N/A	48% (23/48)	25% (13/53)
Number of autoantibodies:			
**≤1 AAb**	N/A	31% (15/48)	30% (16/53)
**≥2 AAb**	N/A	69% (33/48)	70% (37/53)
Clinical variables at diagnosis:			
**Plasma glucose (mmol/L), mean ± SD**	N/A	N/A	24.3 ± 10.7 (n=43)
**HbA1c (mmol/mol), mean ± SD**	N/A	N/A	79.0 ± 30.1 (n=31)
**Blood pH, mean ± SD**	N/A	N/A	7.34 ± 0.11 (n=42)
**Beta-hydroxybutyrate (mmol/L), mean ± SD**	N/A	N/A	2.83 ± 2.79 (n=44)

*individuals, who have progressed from AAb^+^ stage to clinical T1D during follow-up, ^†^not applicable.

Plasma samples were collected between May 2013 and January 2016 for the pediatric cohort at the DIPP study center in Turku, Finland, and between February 2012 and March 2020 for the adult cohort at the University of Eastern Finland, Kuopio, Finland. Plasma was collected from heparinized peripheral blood samples after centrifugation at 700 x g for 10 min and stored at -80°C until analysis.

All participants and/or their legal guardians provided written informed consent, as mandated by the Declaration of Helsinki. The study was approved by local ethics committees of the University Hospitals of Turku and Kuopio, and for the DIPP study by the ethics committee of the Hospital District of Northern Ostrobothnia.

### 2.2 Quanterix SiMoA assays

The heparin plasma samples were randomized for plating, then thawed and aliquoted in batches to reduce freeze-thaw cycles. For all samples, assessment of IL-21 levels was performed using the Quanterix SiMoA assay as previously reported (20). Assessment of IL-17A, IL-6 and TNF-α levels was performed using the Cytokine 3 Plex B Quanterix SiMoA assay, according to manufacturer’s instructions.

The spike recoveries were determined by comparing the spike samples of the observed back-calculated concentration from calibration curve against known spiked concentration. Limits of detection (LODs) were determined by the selection criteria of 75%–125% of the calibration recovery (i.e., comparing the calibration samples of the calculated concentration to expected concentration). Limits of Quantitation (LOQs) were determined by the selection criteria of 75%–125% of the spike recovery (i.e., comparing the spike samples of the calculated concentration to expected concentration). The analyses for the adult cohort were performed in two batches, with slightly different LOD and Lower Limit of Quantitation (LLOQ) values, and batch correction was calculated using linear model in R (25).

### 2.3 C-peptide and hsCRP measurements

C-peptide was measured from heparinized plasma with electrochemiluminescence immunoassay (detection limit 0.007 nmol/L, Cobas, Roche Diagnostics) and high-sensitivity C-reactive protein (hsCRP) from heparinized plasma with particle enhanced immunoturbidimetric assay (detection limit 0.15 mg/L, Cobas, Roche Diagnostics).

### 2.4 Statistical analyses

Graphpad Prism version 9.1.0 (GraphPad Software, San Diego, California USA) was used for statistical analyses. Kruskal-Wallis test followed by Dunn’s multiple comparison test was applied when comparing more than two groups. Mann-Whitney test or Wilcoxon matched-pairs signed-rank test was used when comparing two groups. No power calculations were made as the study was exploratory in nature with very limited previous data to support the calculations, and sample sizes were determined in part by feasibility. Spearman’s correlation was calculated when relationships between plasma cytokine concentrations and different variables were assessed. Simple linear regression was used to calculate regression lines for each group. Slopes and intercepts of the regression lines were compared, and two-tailed P-values were calculated for them in Prism. P-values <0.05 were considered to indicate statistical significance.

## 3 Results

### 3.1 Plasma IL-21 levels are higher in adults with established type 1 diabetes than in healthy controls

**Figure 1:**
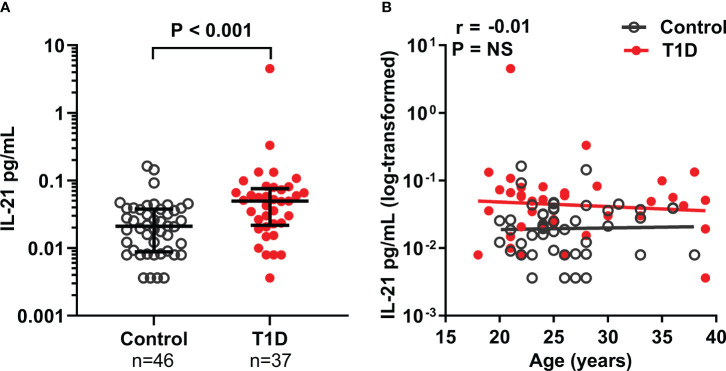
Plasma IL-21 levels are elevated in adults with T1D. **(A)** Plasma IL-21 levels in adults with established T1D and healthy controls. Mann-Whitney U-test was used for statistical analysis. **(B)** Correlation between age and log_10_-transformed plasma IL-21 levels was examined using Spearman’s correlation by pooling the data from both study groups and is expressed together with the P value on the plot. The elevations of the linear regression lines were significantly different between the study groups (P = 0.001). Medians and IQRs are shown in the **(A)**.

Plasma IL-21, as well as IL-17A, TNF-α, and IL-6 levels were analyzed in 37 adults with established T1D and in 47 age-matched healthy controls. Plasma IL-21 levels were 2.5-fold higher in T1D patients (median 0.05 pg/mL) compared to healthy controls (median 0.02 pg/mL, P < 0.001, **Figure 1A**). Plasma IL-6 levels were also elevated in T1D patients compared to controls (median 1.03 pg/mL vs. 0.61 pg/mL, P < 0.05), but plasma IL-17A and TNF-α levels were similar between the study groups (**Supplementary Figure 1**).

Next, we examined whether selected clinical variables were associated with plasma IL-21 levels. No correlations were observed between age, hsCRP, C-peptide levels, HbA1c values, body-mass index (BMI), or disease duration with plasma IL-21 levels in the T1D patients (**Figure 1B**, **Supplementary Figure 1**). However, plasma IL-21 levels correlated positively with both TNF-α (P < 0.001, r=0.40) and IL-6 (P < 0.05, r=0.27) levels. Moreover, IL-17A levels correlated with TNF-α (P < 0.001, r=0.57) and IL-6 (P < 0.001, r=0.60) levels, while TNF-α levels correlated with hsCRP (P < 0.05, r=0.26) (**Supplementary Figure 1**).

### 3.2 Plasma IL-21 levels are similar in children with newly diagnosed type 1 diabetes, children at-risk for type 1 diabetes and healthy controls

**Figure 2:**
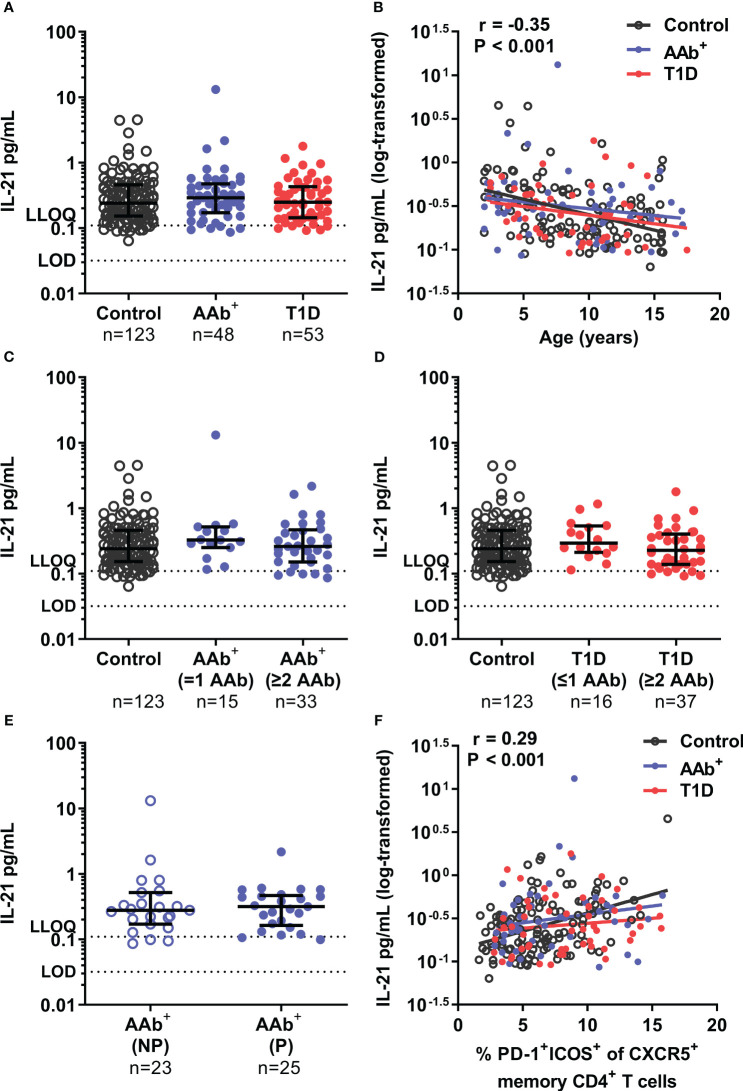
Similar plasma IL-21 levels in children with newly diagnosed T1D, AAb+ at-risk children and healthy children. **(A)** Plasma IL-21 levels in children with newly diagnosed T1D, AAb^+^ at-risk children and control children. **(B)** Correlation between age and log_10_-transformed plasma IL-21 levels was examined using Spearman’s correlation by pooling the data from all study groups and is presented together with the P value on the plot. The elevations of the linear regression lines were comparable between the study groups. AAb^+^ at-risk children **(C)** and children with newly diagnosed T1D **(D)** were stratified into two subgroups based on the number of persistent autoantibodies detected at sampling (positive for ≤1 autoantibodies and ≥2 autoantibodies). **(E)** AAb^+^ at-risk children were divided into non-progressors (NP) and progressors (P) depending on whether they had later progressed to T1D during follow-up. Kruskal-Wallis with Dunn’s multiple comparisons test or Mann-Whitney U-test was used for statistical analyses. Medians with IQRs are shown, and limit of detection (LOD) and lower limit of quantification (LLOQ) are represented with dotted lines in A, C–E. **(F)** Correlation between the frequency of circulating activated Tfh (PD-1^+^ICOS^+^ of CXCR5^+^ memory CD4^+^ T cells) and log_10_-transformed plasma IL-21 levels was examined using Spearman’s correlation by pooling the data from all study groups, and is expressed together with the P value on the plot. The elevations of the linear regression lines were comparable between the study groups.

Next, we analyzed the plasma cytokine levels in samples from 53 children with newly diagnosed T1D and 48 AAb^+^ at-risk children, as well as from 123 healthy age-matched controls. In the pediatric cohort, plasma IL-21 levels (**Figure 2A**), as well as IL-17A, TNF-α, and IL-6 levels **Supplementary Figure 2**) were similar between children with T1D, AAb^+^ at-risk children, and healthy controls. Of note, plasma IL-21 levels in children (median 0.26 pg/mL for the whole pediatric cohort) were around ten-fold higher compared to those observed in the adult cohort (**Figures 1A**, **2A**). Accordingly, a strong negative correlation between age and IL-21 levels (P < 0.001, r=-0.35), as well as to a lesser extent between age and IL-17A (P < 0.01, r=-0.20) and TNF-α (P < 0.001, r=-0.24) levels, was observed in the pediatric cohort (**Figure 2B**, **Supplementary Figure 2**). However, even after stratification with age, no differences in plasma IL-21 levels between the pediatric study groups were detected (**Figure 2B**, **Supplementary Figure 2**). Finally, these results were further corroborated by a stringent pairwise analysis of a subset of samples from T1D and AAb^+^ children that were drawn and processed in parallel with a sample from an age-matched healthy control child on the same day (**Supplementary Figure 3**). In addition, we analyzed correlation between selected clinical variables and plasma cytokine levels in children with newly diagnosed T1D. We did not observe correlations between cytokine levels and clinical variables at diagnosis (plasma glucose, HbA1c, blood pH, or beta-hydroxybutyrate levels) (**Supplementary Figure 2**). Of note, similar to the adult cohort, IL-17A levels correlated with TNF-α (P < 0.001, r=0.66) and IL-6 (P < 0.001, r=0.48) levels also in the pediatric cohort (**Supplementary Figure 2**).

We have previously observed that the frequency of both circulating Tfh (6) and Tph (14) cells was increased in children with newly diagnosed T1D positive for at least two AAbs but not in those positive for one AAb or none. Hence, we next stratified both AAb^+^ children and children with T1D into two subgroups based on the number of autoantibodies detected (≤1 or ≥2) in these children. However, plasma IL-21, as well as IL-17A, TNF-α, and IL-6 levels were again similar in AAb^+^ children and children with T1D positive for either ≤1 or ≥2 autoantibodies (**Figures 2C, D**, **Supplementary Figure 4**). In addition, we analyzed whether plasma cytokine levels differed between those AAb^+^ at-risk children who had progressed to T1D (mean time to T1D after sampling 2.6 ± 1.4 years) and those who had not. However, again, no differences were detected between the groups (**Figure 2E**, **Supplementary Figure 5**).

Finally, flow cytometry data were available on selected T-cell subset frequencies for the pediatric samples (6, 26). Hence, we examined the correlation between plasma IL-21 levels and selected T-cell subset frequencies. The frequencies of activated Tfh cells (PD-1^+^ICOS^+^ of CXCR5^+^ memory CD4^+^ T cells) (6) positively correlated with plasma IL-21 levels (P < 0.001, r=0.29, **Figure 2F**). A weaker positive correlation was also observed with the frequency of regulatory T cells (Treg; CD25^+^CD127^low^ of CD4^+^ T cells) (26) and plasma IL-21 levels (P < 0.05, r=0.17, **Supplementary Figure 6**). However, no correlation was observed between the frequency of Th17 cells (CCR6^+^CXCR3^-^ of memory CD4^+^ T cells) (26) and plasma IL-21 levels (**Supplementary Figure 6**).

## 4 Discussion

In this study, we demonstrated that adults with established T1D had significantly elevated plasma IL-21 and IL-6 levels compared to age-matched controls. IL-21 levels did not correlate with the time from diagnosis or C-peptide levels, a marker of residual beta-cell function. Moreover, no correlation between plasma IL-21 levels and HbA1c levels, a marker related to glycemic control, or hsCRP, a marker related to inflammation, was observed. Therefore, the elevated plasma IL-21 levels do not appear to be associated with the extent of beta-cell destruction, hyperglycemia itself, or with underlying inflammation in T1D patients in the cohort studied.

In the pediatric cohort, we did not observe differences in plasma IL-21 levels between patients with newly diagnosed T1D, AAb^+^ at-risk children, and healthy age-matched children. Similar to adults, no correlation between plasma IL-21 levels and clinical variables, such as level of hyperglycemia (blood glucose and HbA1c levels at diagnosis) or markers of ketoacidosis (blood pH and beta-hydroxybutyrate levels) were found in children with T1D. Moreover, no associations between plasma IL-21 levels and autoantibody status or the risk of progression to clinical T1D in AAb^+^ at-risk children were observed.

An important observation in our study was that plasma IL-21 levels appear to be physiologically around ten-fold higher in children than in adults. Importantly, for the other cytokines, IL-17A, TNF-α, and IL-6, assessed in this study, no such phenomenon was observed. This observation could potentially explain the discrepancy that higher plasma IL-21 levels were observed only in adult T1D patients but not in children with T1D. As the plasma IL-21 levels were only modestly elevated in adult T1D patients compared to controls, a corresponding small change would be masked by the considerably higher physiological background levels in children. Another interesting finding in the pediatric cohort was that plasma IL-21 levels correlated with the frequency of activated Tfh cells in blood, potentially implicating Tfh cells as a major source of plasma IL-21.

Three previous studies have reported slightly elevated plasma IL-21 levels in both adult and pediatric patients with T1D (21, 22), as well as in AAb^+^ at-risk children (23). However, in these studies either ELISA or Luminex xMAP technologies were used, and the IL-21 levels reported were >100-fold higher than in our study using the ultrasensitive Quanterix SiMoA technology. Previous validation results strongly suggest that these conspicuously higher IL-21 levels reported in the previous studies may not accurately reflect physiological cytokine levels, as these older methods lack both the sensitivity and specificity required to detect endogenous IL-21 levels reliably (20).

In addition to using an ultrasensitive analysis method, the major strengths of our study are the large cohort sizes and the stringent matching with HLA background, age, and sampling date in the pediatric cohort, which all considerably strengthen the validity of our results. One caveat of our study is that we were only able to analyze cross-sectional cohorts. In the future, longitudinal analyses could potentially better detect subtle intraindividual alterations in cytokine levels occurring during T1D progression. Moreover, the analysis of patients with type 2 diabetes could help confirm whether the increased plasma IL-21 levels in adults with T1D are truly associated with autoimmunity and are not secondary to hyperglycemia and/or exogenous insulin use.

In conclusion, for the first time, IL-21 was quantified using an ultrasensitive and specific method and found to be elevated in adults with established T1D, supporting a potential role of IL-21 in the T1D disease process. In contrast, in the pediatric cohort, comprising children with newly diagnosed T1D, AAb^+^ children at-risk for T1D, and age-matched healthy controls, we did not observe differences in plasma IL-21 levels between the study groups. The physiologically higher plasma IL-21 levels in children may, however, mask potential changes caused by T1D autoimmunity. Taken together, the direct detection of plasma IL-21 levels, even by the ultrasensitive method capable of detecting fg/mL concentrations of cytokines employed here, may have limited potential as a biomarker of T1D progression, particularly in children.

## Data availability statement

The raw data supporting the conclusions of this article will be made available by the authors, without undue reservation.

## Ethics statement

The studies involving human participants were reviewed and approved by local ethics committees of the University Hospitals of Turku and Kuopio and the ethics committee of the Hospital District of Northern Ostrobothnia. Written informed consent to participate in this study was provided by the participants or participants’ legal guardian/next of kin.

## Author contributions

JPo, SM and SB measured the cytokines. RR, JPi, KN-S and JT provided the clinical research samples. MK and RV were responsible for the islet autoantibody analyses of the pediatric cohort. JI was responsible for the HLA screening of the pediatric study subjects. A-MS, JPo, SM, SB, LZ, RJB and TK analyzed the data and drafted the manuscript. TK is the guarantor of this work and, as such, had full access to all the data in the study and takes responsibility for the integrity of the data and the accuracy of the data analysis. All authors contributed to the article and approved the submitted version.
