# Supplementary material for: Evaluation of plasma IL-21 as a potential biomarker for type 1 diabetes progression
Source: Front Immunol. 2023 Jun 21;14:1157265. doi: 10.3389/fimmu.2023.1157265 (PMC10321755; doi:10.3389/fimmu.2023.1157265)
Supplement: Supplementary file 1 [file DataSheet_1.pdf]

frontiers

Supplementary Material

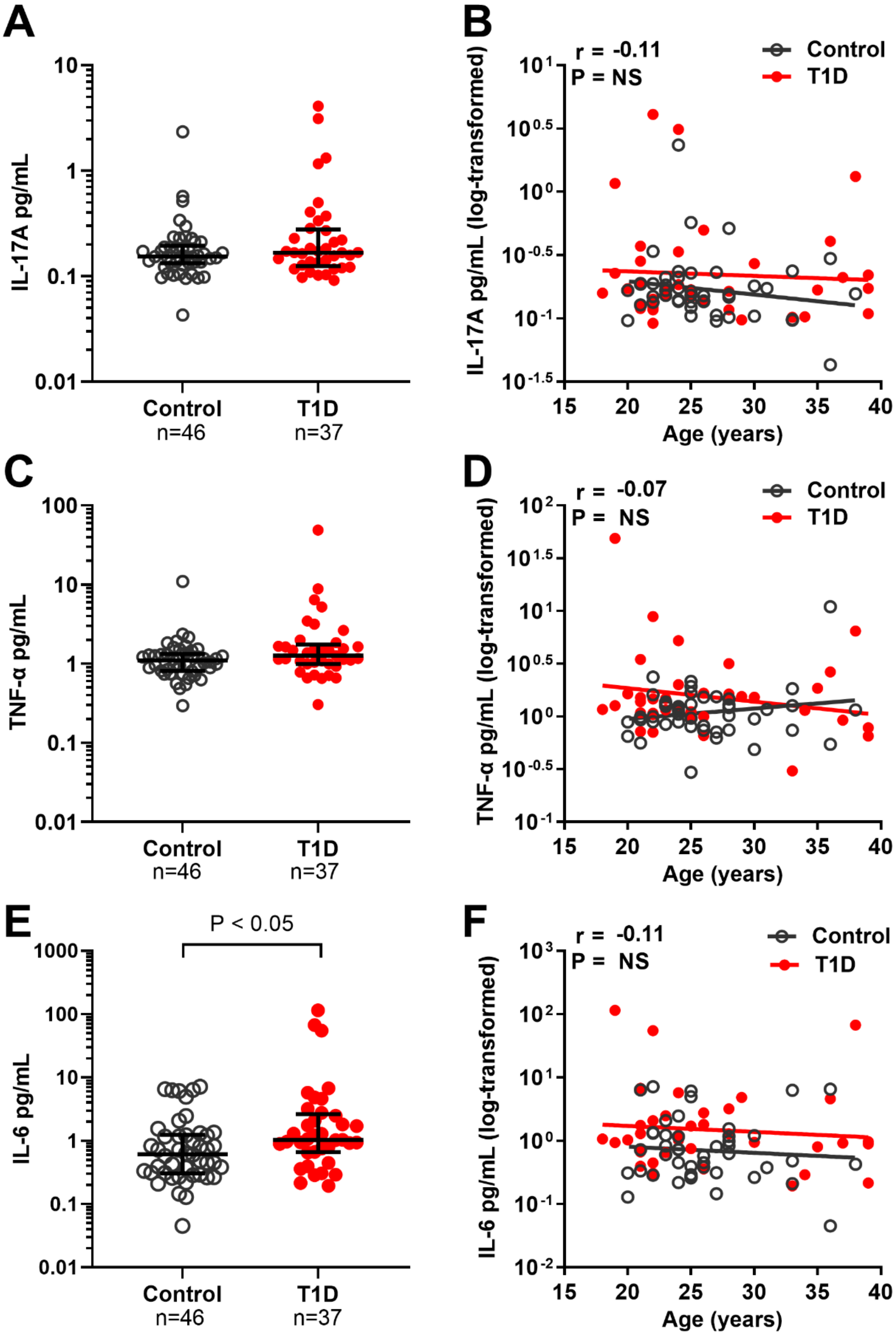

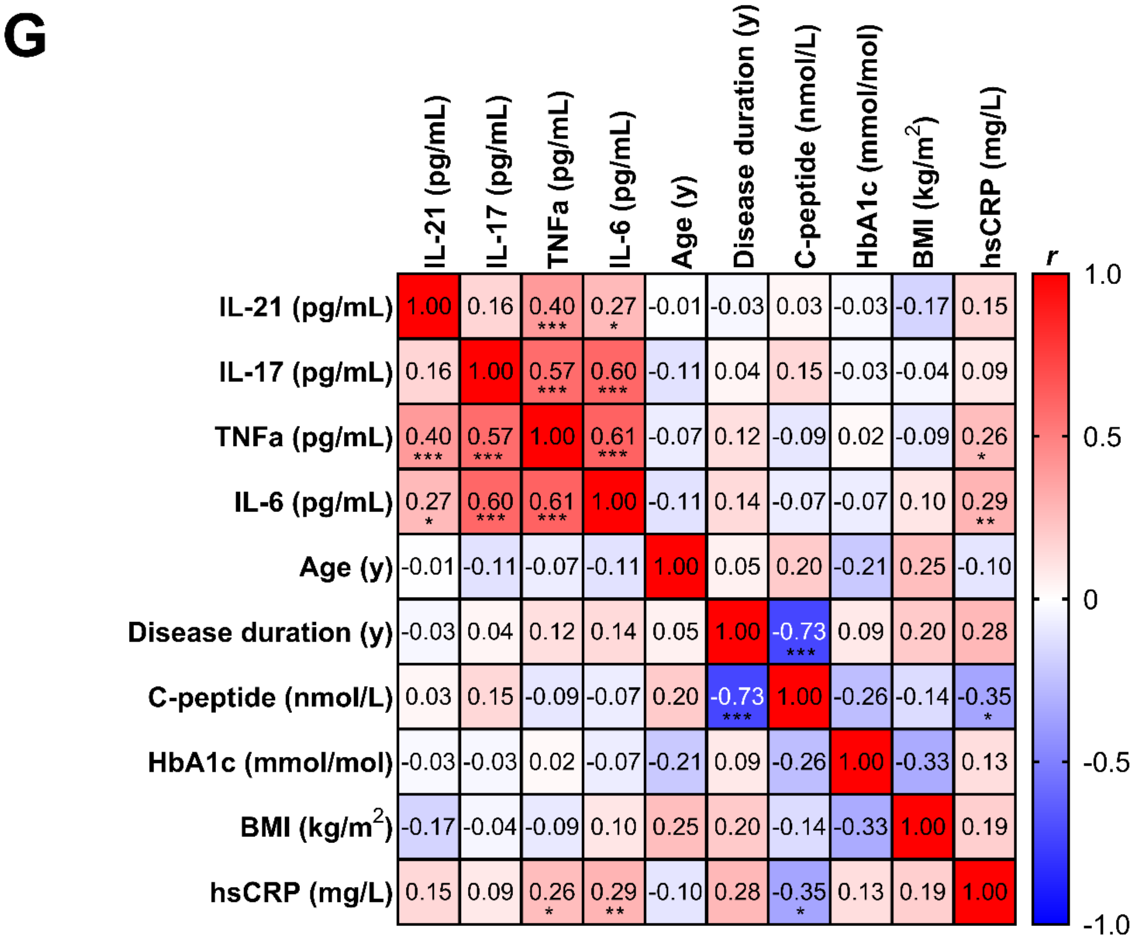

	IL-21 (pg/mL)	IL-17 (pg/mL)	TNFa (pg/mL)	IL-6 (pg/mL)	Age (y)	Disease duration (y)	C-peptide (nmol/L)	HbA1c (mmol/mol)	BMI (kg/m²)	hsCRP (mg/L)
IL-21 (pg/mL)	1.00	0.16	0.40 ***	0.27 *	-0.01	-0.03	0.03	-0.03	-0.17	0.15
IL-17 (pg/mL)	0.16	1.00	0.57 ***	0.60 ***	-0.11	0.04	0.15	-0.03	-0.04	0.09
TNFa (pg/mL)	0.40 ***	0.57 ***	1.00	0.61 ***	-0.07	0.12	-0.09	0.02	-0.09	0.26 *
IL-6 (pg/mL)	0.27 *	0.60 ***	0.61 ***	1.00	-0.11	0.14	-0.07	-0.07	0.10	0.29 **
Age (y)	-0.01	-0.11	-0.07	-0.11	1.00	0.05	0.20	-0.21	0.25	-0.10
Disease duration (y)	-0.03	0.04	0.12	0.14	0.05	1.00	-0.73 ***	0.09	0.20	0.28
C-peptide (nmol/L)	0.03	0.15	-0.09	-0.07	0.20	-0.73 ***	1.00	-0.26	-0.14	-0.35 *
HbA1c (mmol/mol)	-0.03	-0.03	0.02	-0.07	-0.21	0.09	-0.26	1.00	-0.33	0.13
BMI (kg/m²)	-0.17	-0.04	-0.09	0.10	0.25	0.20	-0.14	-0.33	1.00	0.19
hsCRP (mg/L)	0.15	0.09	0.26 *	0.29 **	-0.10	0.28	-0.35 *	0.13	0.19	1.00

Supplementary Figure 1. Plasma IL-17A, TNF-α and IL-6 levels in adults with established T1D and healthy controls. Plasma levels of **(A)** IL-17A, **(C)** TNF-α and **(E)** IL-6 in adults with T1D and healthy controls. Mann-Whitney U-test was used for statistical analysis. Correlation between age and $\log_{10}$-transformed plasma cytokine levels of **(B)** IL-17A, **(D)** TNF-α and **(F)** IL-6 were examined using Spearman's correlation by pooling the data from adult T1D and control groups, and is expressed together with the P value on the plot. Linear regression lines were calculated for each study group. The elevations of linear regression lines differed significantly between study groups for TNF-α ($P = 0.0318$) and IL-6 levels ($P = 0.0103$). **(G)** Spearman's correlation matrix was calculated to examine the correlations between plasma cytokine levels, age, disease duration, as well as C-peptide, HbA1c, BMI and hsCRP values. Numbers within each square represent Spearman r values. T1D patients and healthy controls were pooled in the correlation analyses of the cytokine levels, age and hsCRP. Disease duration, C-peptide, HbA1c and BMI were analyzed only within T1D patients. Medians with IQRs are shown in the panels A, C and E. $*P < 0.05$, $**P < 0.01$, $***P < 0.001$

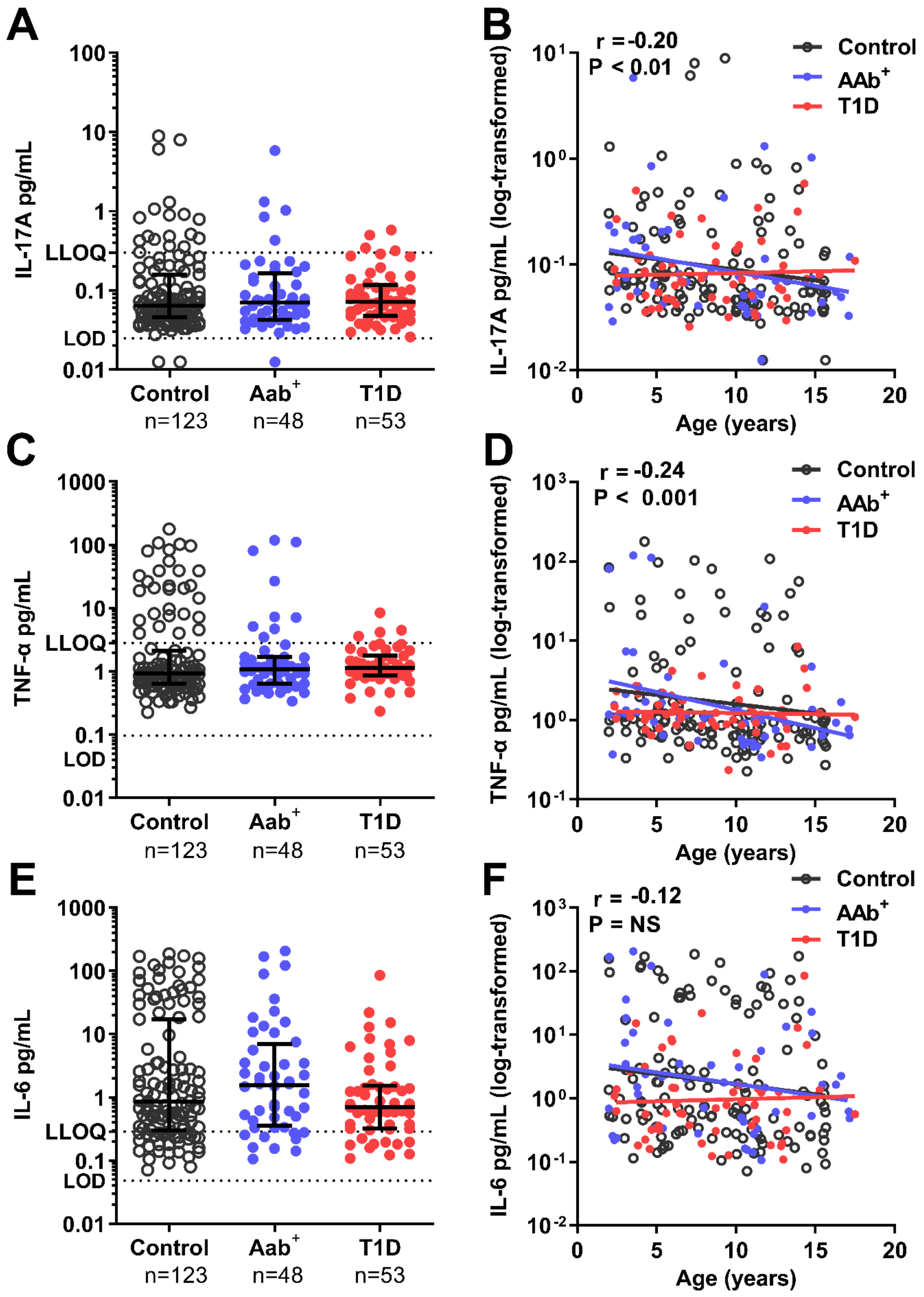
A
IL-17A pg/mL
100
10
1
LLOQ
0.1
LOD
0.01
Control
n=123
Aab+
n=48
T1D
n=53
B
r = -0.20
P < 0.01
Control
AAb+
T1D
IL-17A pg/mL (log-transformed)
Age (years)
C
TNF-α pg/mL
1000
100
10
LLOQ
1
0.1
LOD
0.01
Control
n=123
Aab+
n=48
T1D
n=53
D
r = -0.24
P < 0.001
Control
AAb+
T1D
TNF-α pg/mL (log-transformed)
Age (years)
E
IL-6 pg/mL
1000
100
10
1
LLOQ
0.1
LOD
0.01
Control
n=123
Aab+
n=48
T1D
n=53
F
r = -0.12
P = NS
Control
AAb+
T1D
IL-6 pg/mL (log-transformed)
Age (years)

G

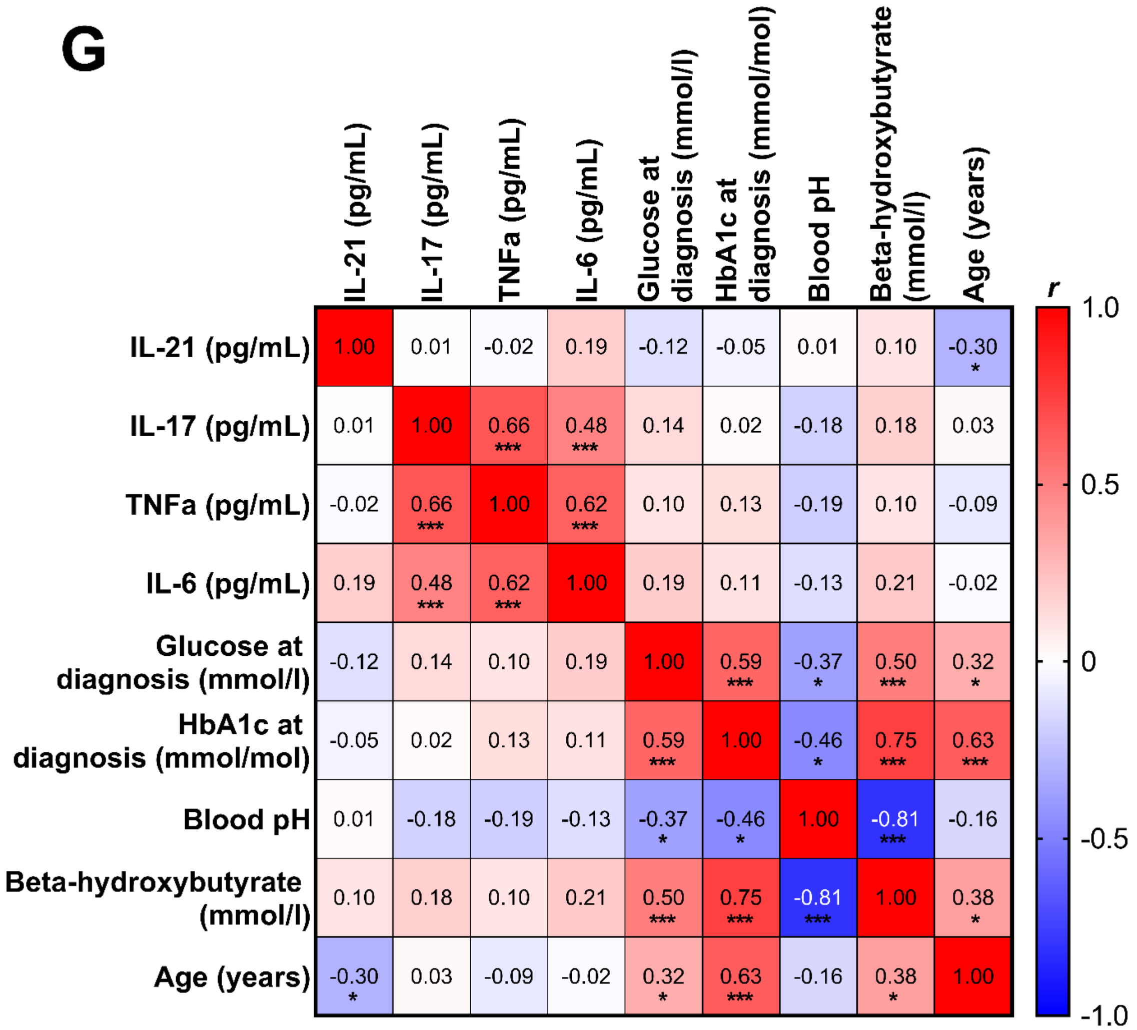

Supplementary Figure 2. Plasma IL-17A, TNF-α and IL-6 levels in children with newly diagnosed T1D, AAb$^+$ at-risk children and healthy children. Plasma **(A)** IL-17A (pg/mL), **(C)** TNF-α and **(E)** IL-6 levels in children with newly diagnosed T1D, AAb$^+$ at-risk children and healthy children. Kruskal-Wallis with Dunn's multiple comparison test was used for statistical analysis. Correlation between $\log_{10}$-transformed plasma **(B)** IL-17A, **(D)** TNF-a and **(F)** IL-6 levels and age was examined using Spearman's correlation by pooling the data from all three study groups, and is expressed together with the P value on the plot. Linear regression lines were calculated for each study group. Elevations of the linear regression lines did not differ between the study groups for any of the cytokines. Medians with IQRs are shown. LOD and LLOQ are represented with dotted lines in panels A, C and E. **(G)** Spearman's correlation matrix was calculated to examine the correlations between different plasma cytokine levels, and plasma glucose, HbA1c, blood pH, beta-hydroxybutyrate, and age at T1D diagnosis. Numbers within each square represent Spearman r values. Pediatric study groups were pooled in the correlation analyses of the cytokine levels. Plasma glucose, HbA1c, blood pH, and beta-hydroxybutyrate at diagnosis were analyzed only among children with newly diagnosed T1D. *$P < 0.05$, **$P < 0.01$, ***$P < 0.001$

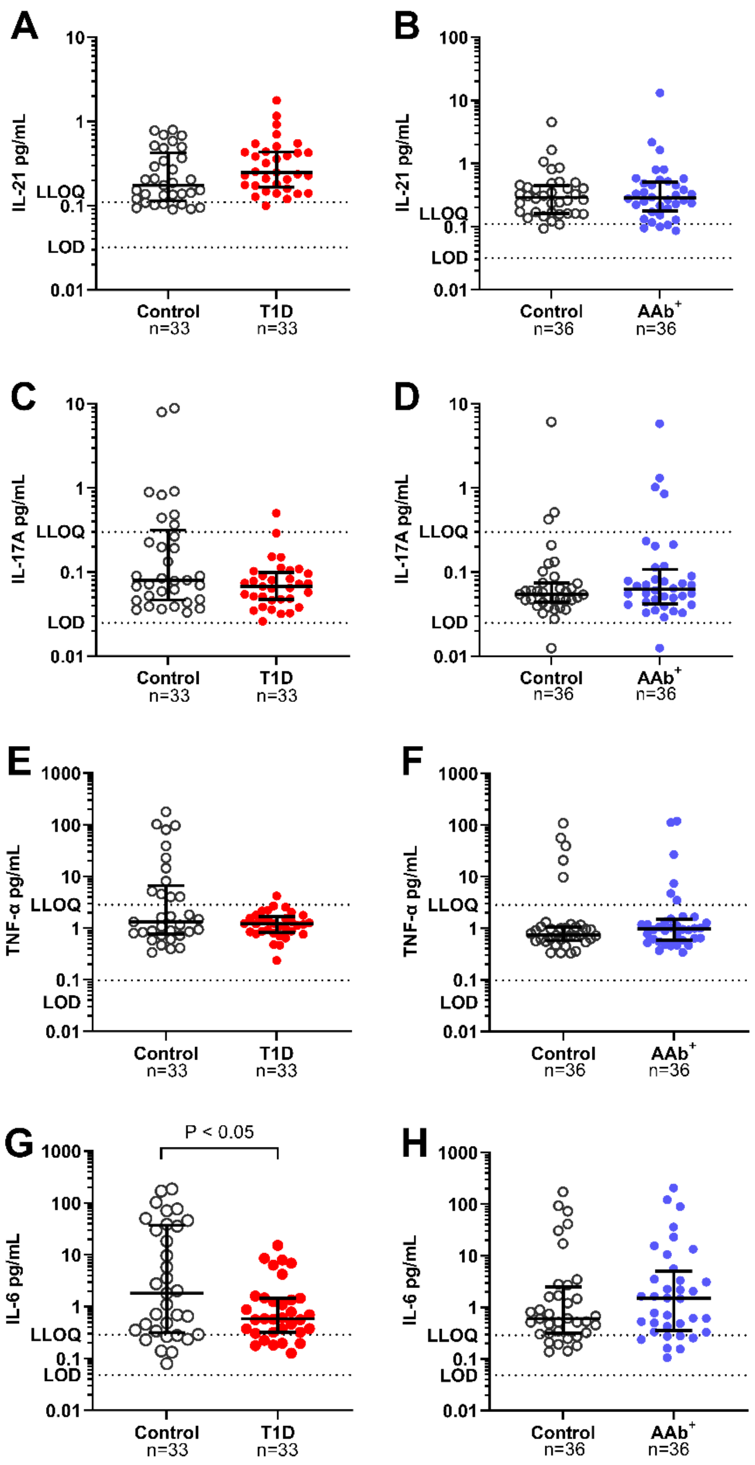

Supplementary Figure 3. Pairwise analyses of plasma IL-21, IL-17A, TNF-α and IL-6 levels in stringently paired case-control pairs. Plasma IL-21, IL-17A, TNF-α and IL-6 (pg/mL) levels in children with newly diagnosed T1D **(A, C, E and G)**, AAb^+ at-risk children **(B, D, F and H)** and healthy children. For the case-control pairs, blood samples were drawn and processed on the same day. Wilcoxon matched-pairs signed-rank test was used for statistical analysis. LOD and LLOQ are represented in dotted lines in each figure.

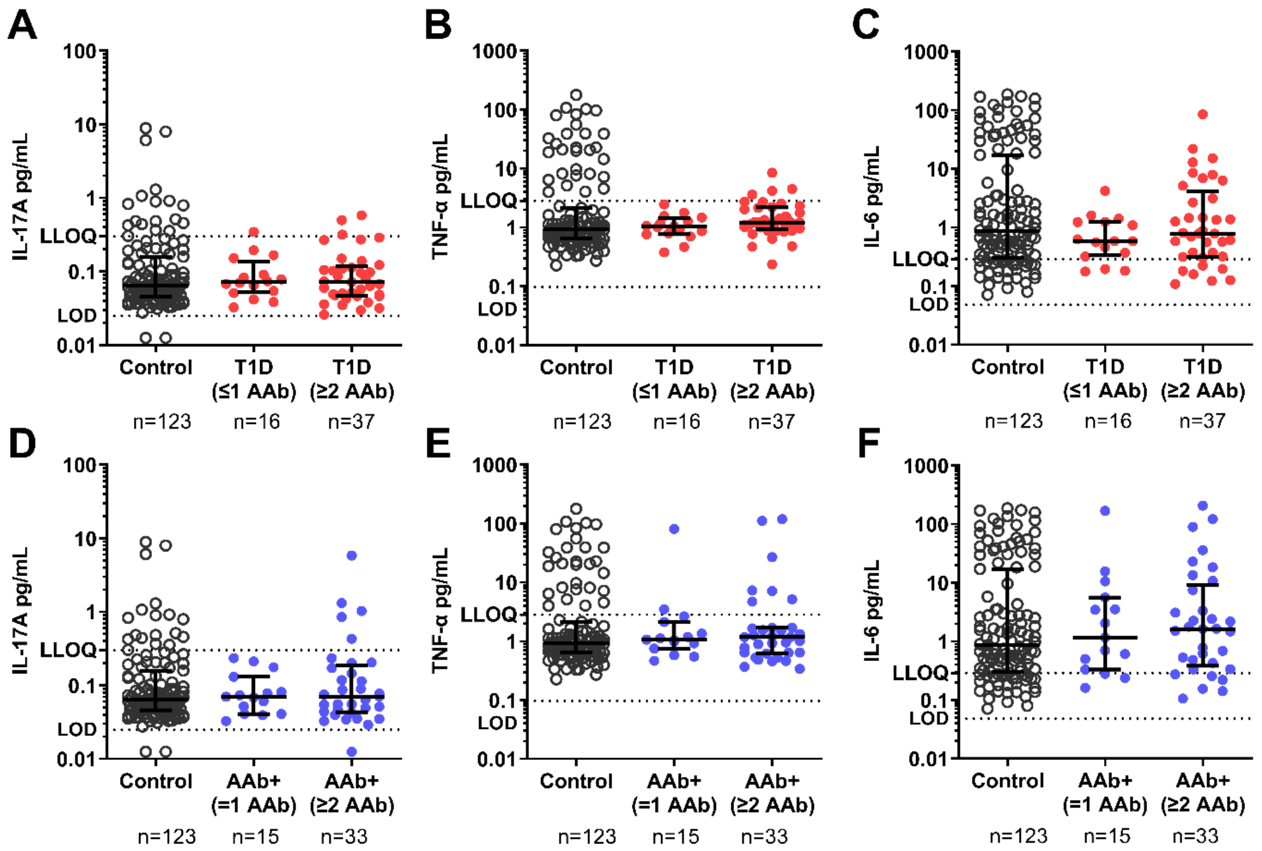

Supplementary Figure 4. Similar plasma IL-17A, TNF-α and IL-6 levels in children with one or multiple autoantibodies. **(A–C)** Children with newly diagnosed T1D and **(D–F)** AAb^+ at-risk children were divided into two groups (positive for ≤1 autoantibodies and ≥2 autoantibodies), according to the number of persistent autoantibodies detected. Kruskal-Wallis test with Dunn's multiple comparisons test was used for statistical analysis. Medians with IQRs are shown. LOD and LLOQ are represented in dotted lines in each figure.

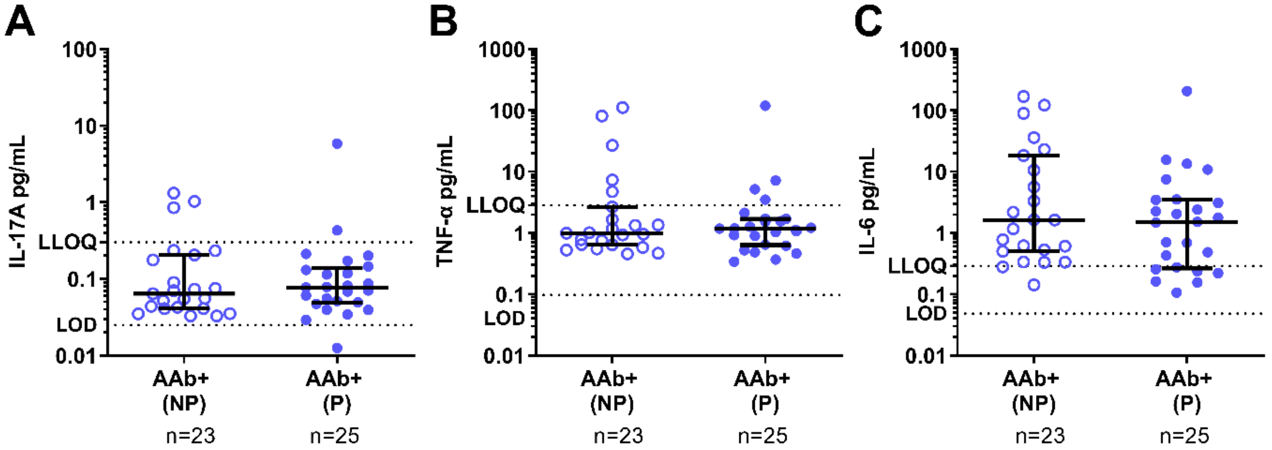

Supplementary Figure 5. Progressor and non-progressor AAb$^+$ at-risk children have similar plasma IL-17A, TNF-α and IL-6 levels. Plasma **(A)** IL-17A, **(B)** TNF-α and **(C)** IL-6 levels in AAb$^+$ at-risk children who were divided into non-progressors (NP) and progressors (P), depending on whether they had later progressed to clinical T1D during follow-up. Mann-Whitney test was used for statistical analysis. Medians with IQRs are shown. LOD and LLOQ are represented in dotted lines in each figure.

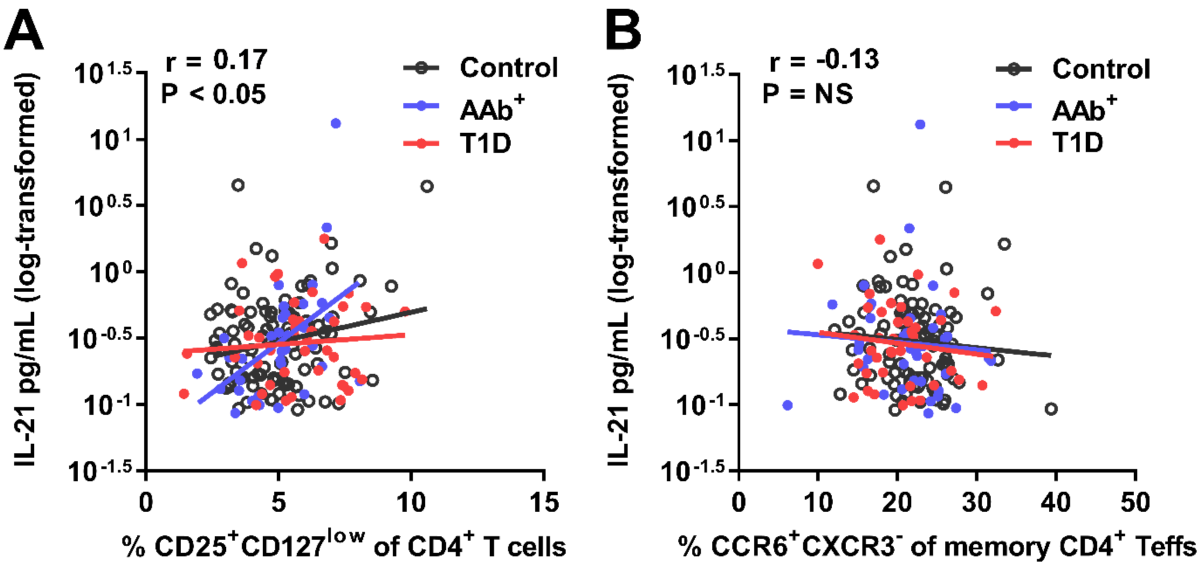

Supplementary Figure 6. The frequency of regulatory T cells correlates with plasma IL-21 levels. Correlation between log_{10}-transformed IL-21 (pg/mL) and **(A)** Treg ($CD25^{+}CD127^{low}$ of $CD4^{+}$ T cells) and **(B)** Th17 ($CCR6^{+}CXCR3^{-}$ of memory $CD4^{+}$ T cells) cell frequencies in PBMCs were examined using Spearman's correlation by pooling all pediatric study groups and is expressed together with the P value on the plot. Linear regression lines were calculated for each study group. Elevations of the linear regression lines did not differ between the study groups
